# Supplementary material for: Evaluation of 16S rDNA Heart Tissue PCR as a Complement to Blood Cultures for the Routine Etiological Diagnosis of Infective Endocarditis
Source: Diagnostics (Basel). 2021 Jul 30;11(8):1372. doi: 10.3390/diagnostics11081372 (PMC8394467; doi:10.3390/diagnostics11081372)
Supplement: Supplementary file 1 [file diagnostics-11-01372-s001.zip › diagnostics-1299563-supplementary.pdf]

Table S1. Detailed microbiological results of patients diagnosed of infective endocarditis

Blood cultures	Heart valve or vegetation cultures	16S rDNA heart tissue PCR	Number of cases
Staphylococcus aureus			**48**
Staphylococcus aureus	Not performed	Not performed	24
Staphylococcus aureus	Negative	Not performed	2
Staphylococcus aureus	*Staphylococcus aureus*	Not performed	5
Negative	*Staphylococcus aureus*	Not performed	2
Staphylococcus aureus	*Staphylococcus aureus*	Negative	1
Staphylococcus aureus	Negative	Negative	5
Staphylococcus aureus	*Cutibacterium acnes*	Negative	1
Staphylococcus aureus	Negative	*Staphylococcus aureus*	6
Staphylococcus aureus	*Staphylococcus aureus*	*Staphylococcus aureus*	1
Negative	Negative	*Staphylococcus aureus*	1
**Coagulase-negative staphylococci (CoNS)**			**69**
Staphylococcus epidermidis	Not performed	Not performed	29
Staphylococcus epidermidis	Negative	Not performed	3
Staphylococcus epidermidis	*Staphylococcus epidermidis*	Not performed	9
Negative	*Staphylococcus epidermidis*	Not performed	1
Staphylococcus epidermidis	Negative	Negative	1
Staphylococcus epidermidis	*Staphylococcus epidermidis*	*Staphylococcus epidermidis*	6
Staphylococcus epidermidis	Negative	*Staphylococcus epidermidis*	6
Negative	Negative	*Staphylococcus epidermidis*	1
Staphylococcus lugdunensis	Not performed	Not performed	2
Negative	Negative	*Staphylococcus lugdunensis*	1
Staphylococcus lugdunensis	Negative	*Staphylococcus lugdunensis*	1
Staphylococcus lugdunensis	*Staphylococcus lugdunensis*	Not performed	1
Staphylococcus auricularis	Negative	Not performed	1
Negative	*Staphylococcus auricularis*	Not performed	1
Staphylococcus hominis	Not performed	Not performed	2
Negative	*Staphylococcus haemolyticus*	Not performed	1
Staphylococcus warneri	*Staphylococcus warneri and Cutibacterium acnes*	Negative	1
Other CoNS	Not performed	Not performed	2
**Viridans group streptococci**			**49**
Streptococcus mitis	Negative	*Streptococcus mitis*	1
Negative	Negative	*Streptococcus mitis*	2
Streptococcus mutans	Negative	*Streptococcus mutans*	1
Streptococcus mutans	*Staphylococcus haemolyticus*	*Streptococcus mutans*	1
Negative	Negative	*Streptococcus mutans*	1
Streptococcus anginosus	*Streptococcus anginosus*	Not performed	1
Streptococcus anginosus	Not performed	Not performed	1
Streptococcus constellatus	Negative	Negative	1
Streptococcus gallolyticus	Not performed	Not performed	8
Streptococcus gallolyticus	Negative	*Streptococcus gallolyticus*	5
Streptococcus gallolyticus	Negative	Not performed	1

Streptococcus gallolyticus	*Streptococcus gallolyticus*	Not performed	2
Streptococcus gallolyticus	Negative	Negative	1
Streptococcus gordonii	Not performed	Not performed	3
Streptococcus gordonii	Negative	Not performed	1
Streptococcus gordonii	Negative	*Streptococcus gordonii*	2
Streptococcus oralis	Not performed	Not performed	1
Streptococcus oralis	Negative	Not performed	1
Streptococcus oralis	Negative	Negativo	1
Streptococcus oralis	Negative	*Streptococcus oralis*	2
Streptococcus oralis	*Cutibacterium acnes*	*Streptococcus oralis*	1
Streptococcus parasanguis	Not performed	Not performed	2
Streptococcus salivarius	Negative	Negative	1
Streptococcus sanguinis	Not performed	Not performed	3
Streptococcus sanguinis	Negative	Negative	1
Streptococcus sanguinis	Negative	*Streptococcus sanguinis*	2
Other viridans streptococci	Negative	Negative	1
Other viridans streptococci	Not performed	Not performed	1
**Beta-hemolytic streptococci**			**5**
Streptococcus dysgalactiae	*Streptococcus dysgalactiae* and *Staphylococcus aureus*	Not performed	1
Streptococcus agalactiae	Not performed	Not performed	2
Negative	Negative	*Streptococcus agalactiae*	1
Streptococcus agalactiae	Negative	*Streptococcus agalactiae*	1
***Enterobacterales* and nonfermenting Gram negative bacilli**			**8**
Escherichia coli	Negative	Not performed	1
Negative	Negative	*Escherichia coli*	1
Escherichia coli	Negative	*Escherichia coli*	1
Enterobacter cloacae	Not performed	Not performed	1
Negative	Negative	*Chryseobacterium* spp.	1
Pseudomonas aeruginosa	*Pseudomonas aeruginosa*	Not performed	1
Pseudomonas aeruginosa	Not performed	Not performed	1
Serratia marcescens	*Serratia marcescens*	Not performed	1
**Enterococci**			**46**
Enterococcus faecalis	*Enterococcus faecalis*	Not performed	5
Enterococcus faecalis	Negative	Not performed	4
Enterococcus faecalis	Not performed	Not performed	10
Negative	*Enterococcus faecalis*	Not performed	1
Enterococcus faecalis	*Enterococcus faecalis*	*Enterococcus faecalis*	7
Enterococcus faecalis	Negative	*Enterococcus faecalis*	7
Enterococcus faecalis	*Staphylococcus cohnii*	*Enterococcus faecalis*	1
Enterococcus faecalis	Negative	Negative	7
Enterococcus faecalis	*Enterococcus faecalis*	Negative	1
Enterococcus faecium	Not performed	Not performed	2
Enterococcus faecium	Negative	*Enterococcus faecium*	1
**Candida**			**2**

Candida metapsilopsis	*Candida metapsilopsis*	Not performed	1
Candida parapsilopsis	Not performed	Not performed	1
**Other**			**17**
Abiotrophia defectiva	Not performed	Not performed	1
Aerococcus urinae	Negative	Negative	1
Agregatibacter actinomycetemcomitans	Not performed	Not performed	1
Agregatibacter actinomycetemcomitans	*Klebsiella pneumoniae*	Not performed	1
Listeria monocytogenes	Not performed	Not performed	1
Negative	Negative	*Tropheryma whipplei*	2
Negative	Negative	*Coxiella burnetii*	4
Cutibacterium acnes	Negative	*Cutibacterium acnes*	1
Negative	*Cutibacterium acnes*	Not performed	1
Negative	*Cutibacterium acnes*	*Cutibacterium acnes*	2
Negative	Negative	*Bartonella henselae*	1
Streptococcus pneumoniae	Negative	Not performed	1
**Polymicrobial infection**			**4**
Negative	*Staphylococcus aureus* and *Proteus mirabilis*	Not performed	1
Candida albicans	*Candida albicans*	*Streptococcus dysgalactiae*	1
Staphylococcus epidermidis	*Staphylococcus epidermidis* and *Staphylococcus hominis*	Negative	1
Granulicatella adiacens	Negative	*Staphylococcus epidermidis*	1
**Negative**			**18**
Negative	Not performed	Not performed	13
Negative	Negative	Negative	4
Negative	Negative	Not performed	1
